# Correction: Functional impact of cardiac implanted devices on ipsilateral shoulder range of motion, scapular mobility, and self-reported quality of life

**DOI:** 10.1371/journal.pone.0348025

**Published:** 2026-04-22

**Authors:** Cansu Cosgun, Muharrem Said Cosgun, Oznur Buyukturan, Buket Buyukturan

The authors, Cansu Cosgun and Muharrem Said Cosgun, should be noted as shared co-corresponding authors on this work. Muharrem Said Cosgun’s email address is: drsaidcosgun2009@hotmail.com.

The ORCID iD of the second author is missing. Please see the author’s ORCID iD here:

Author Muharrem Said Cosgun’s ORCID iD is: 0000-0003-1042-9954 (https://orcid.org/000-0003-1042-9954).
