# Supplementary material for: Functional impact of cardiac implanted devices on ipsilateral shoulder range of motion, scapular mobility, and self-reported quality of life
Source: PLoS One. 2023 Apr 6;18(4):e0284178. doi: 10.1371/journal.pone.0284178 (PMC10079016; doi:10.1371/journal.pone.0284178)
Supplement: Attachment — Submitted filename: Response to Reviewers.docx [file pone.0284178.s001.docx]

Dear Academic Editor (Eyüp Serhat Çalık),

1. We've edited our manuscript to meet PLOS ONE's styling requirements, including file naming.

2. In our Data Availability statement, we have specified where the minimum dataset can be found.

3. We've included our full ethical statement in the "Methods" section of our manuscript file.

4. We remove figure 2 from your submission.

Kind regards.

Dear Reviewer #1,

1. Linguistic errors in the entire document have been corrected according to your suggestions.

2. Added references on LSST and SDT.

3. The possible negative effects of comorbidities such as CAD and CHF on the evaluation parameters are mentioned in the limitations paragrahp.

4. Disadvantages of the cross-sectional design of the study were mentioned in the limitations paragrahp.

Kind regards.

Dear Reviewer #2 (Diane W. Braza, MD),

1. It was defined in the ''Shoulder evaluations'' section where the range of motion measurements was ''active''.

2. The title has been changed as per your suggestion.

Kind regards.
